# Is Sexual Ornamentation an Honest Signal of Male Quality in the Chinese Grouse (*Tetrastes sewerzowi*)?

**DOI:** 10.1371/journal.pone.0082972

**Published:** 2013-12-26

**Authors:** Chen Yang, Jie Wang, Yun Fang, Yue-Hua Sun

**Affiliations:** 1 Key Laboratory of Animal Ecology and Conservation Biology, Institute of Zoology, Chinese Academy of Sciences Institute of Zoology, Chinese Academy of Sciences, Beijing, P. R. of China; 2 Chengdu Institute of Biology, Chinese Academy of Sciences, Chengdu, Sichuan, P. R. of China; Arizona State University, United States of America

## Abstract

We examined the variation in sexual ornamentation of male Chinese grouse (*Tetrastes sewerzowi*) in the Gansu Province, China, seeking to identify factors involved in whether ornament size and brightness are honest signals of male quality. Compared to unmated males, mated males had significantly larger and redder combs and, although they did not have significantly larger territories, they defended them more vigorously. Mated males had significantly higher blood carotenoid and testosterone levels, significantly better body condition, and significantly lower parasite loads than unmated males. Our findings are thus consistent with the hypothesis that comb size and color are honest signals of better male quality in the grouse, mediated through lower parasite loads and/or higher testosterone levels.

## Introduction

Male ornamentation, such as striking plumage, combs, brightly-colored gapes and wattles are important traits in sexual selection. Brightly colored or exaggerated ornaments are reliable signals for advertising individual quality in both male-male competition and mate choice. Females mating with males having more elaborate ornaments will benefit directly from any or all of: greater paternal investment in young, better habitat quality, lower transmission of parasites, and greater breeding success for their male young [1], [2]. On the other hand, females may assess the variation of male genetic quality by condition dependent traits. As a result, females mating with males having such exaggerated ornaments will also gain the indirect benefit of passing the attractive signal to offspring and leaving more descendants. Offspring thus inherit superior traits for both body condition and vitality from their parents [3], [4].

Another question arises, are sexual ornaments honest signals in sexual selection, or can males cheat females by the exaggerating their ornaments? In order to answer this question, at least two hypotheses have been proposed to explain how ornamentation may convey an honest signal of male quality despite the obvious benefit that would accrue to cheaters. According to the parasite-mediated sexual selection hypothesis (PMSSH), individuals with superior ornamentation are those with greater resistance to parasites and therefore able to invest more resources to benefiting females [5]. The immunocompetence handicap hypothesis (ICHH) states that hormones enhance sexual trait expression but impair immunity, and offers a framework for explaining the interaction between the endocrine system and immune function [6]. ICHH incorporates two models: 1) Testosterone is a key factor to regulate the production of male secondary sexual traits and immunosuppression result in greater vulnerability to pathogen or parasite attack. Therefore, only high-quality males can afford to display larger sexually dimorphic ornaments, as they can afford having higher testosterone levels despite a relatively lower cost of immunocompetence [7]. 2) Carotenoids also play an important role as antioxidants and immuno-stimulants [8], [9], as the pigment color of secondary sexual ornaments has a high cost, dependent on carotenoids of limited availability [10]. Carotenoids cannot be synthesized by vertebrates and must be obtained through foraging [11], so males can still afford to allocate carotenoids to sexual ornamentation unless they have genuinely garnered amounts beyond those needed for self-maintenance and the immune function [12], [13]. Moreover, since the allocation of carotenoids is modulated by sex-steroid hormones such as testosterone [14]–[16], males with higher testosterone may have more distinct sexual traits and aggressive behavior and thus more mating success (*e.g*., red grouse *Lagopus lagopus*) [17]; but may also be more prone to injury and increased mortality [18], [19]. Although it is a challenge to the alleged antioxidant benefits of carotenoids [20], it is plausible, under both hypotheses, that individuals of inferior quality are sufficiently penalized for cheating that selection enforces “honesty” in their sexual ornamentation signaling systems.

Numerous laboratory and field investigations have been conducted to test the PMSSH and ICHH hypotheses, especially in passerines, such as the zebra finch (*Taeniopygia guttata*) [8] and the greenfinch (*Carduelis chloris*) [21], [22]. More recently, grouse (Tetraonidae) have been widely investigated [23], [24], undoubtedly because of their variable mating systems and distinct secondary sexual traits [25]. The Chinese grouse (*Tetrastes sewerzowi*), endemic to central China and the smallest and most southerly distributed grouse species in the world [26]. Due to hunting, insecticide application, habitat loss and fragmentation, and human disturbance, the Chinese grouse population have shown a declining trend, and were therefore listed as endangered in the China Red Data Book [27] and Near Threatened by IUCN 2012. This species is monogamous with a breeding-age male-female sex ratio of ca 64∶36, meaning that males must compete for females and ca 44% are unsuccessful [28]. Males with better territories may have more opportunities to pair with females [28], [29], but how they compete for females is poorly known. Given the above, the Chinese grouse is particularly appropriate for such studies.

Although many lines of evidence support the idea that carotenoid-based signals honestly reflect an individual’s health status, territorial aggression and ability to resist parasites [30], few studies have tested the relationship between parasite load, androgens and ornaments, and territorial behavior systematically in the field within a single paper. Previous research has only focused on adult male competition with regards to sexual selection, while the effects of age and sex were not considered. Furthermore, territorial behavior was selected as the only candidate variable for an individual’s territorial aggression, but lacked an estimation of the home range size, especially for the species with a stable territory during the breeding season.

In this study, we investigated whether carotenoid-based ornamentation, both in color and size, predicted body condition, immune responsiveness or parasite load, and the relationships between circulating carotenoids, body condition, immune responsiveness and parasites between age and sex. We sought to test whether male sexual ornamentation and its brightness are honest signals reflecting body condition in the Chinese grouse. We also compared testosterone and carotenoid levels and parasite infection rates in mated and unmated males in an effort to determine which factors modulate sexual ornamentation. Finally, we examined the relationships among two kinds of territorial behaviors (flutter jump and territorial call), parasite abundance, body condition, testosterone concentration, and carotenoid-based ornamentation, and compared the home range size between mated and unmated male grouse, seeking to identify the driving traits for interspecific behavioral differences.

## Materials and Methods

### Ethics Statement

All experimental procedures on animals used in the present study had been given prior approval and were supervised by the Animal Care and Use Committee of the Institute of Zoology, the Chinese Academy of Sciences (Project No. 2008/73). Permits for animal collection and observation were approved by the Department of Wildlife Management, Bureau of Gansu Forestry Administration, Gansu province, China. All staff, fellows and students received appropriate training before performing animal studies. All the radio transmitters were removed after the experiment.

### Study Area

We conducted this study from 2008 to 2009 at the Lianhuashan Natural Reserve in Gansu Province, central China (34°40′67″N, 103°30′84″E). The complete description of climate and vegetative cover for the study area were in the previous published paper [28].

### Study Population

In the spring of 2008 and 2009, we captured forty-eight Chinese grouse (35 males, 13 females) using snare poles, nets, or walk-in traps. Birds were color-banded and equipped with necklace transmitters (Holohil: Model RI-2B) weighing about 12 g, or 3–4% of their body weight (Table S1) [28]. Sex was determined by the presence of the black chin-patch (black chin-patch present in males but not in females) [31], and age was identified from plumage characteristics (adults have a distinct boundary on the tip of the innermost primary feathers, while juveniles have a diffuse boundary) [32]. The number of adult is 24, juveniles is 11. The behavior of all the captured males was monitored to determine their mating status in the breeding season (from March to July). Our work was conducted under the appropriate permits from the local nature reserve and all the radio transmitters were removed after the experiment.

### Blood Measurements

Once a Chinese Grouse was captured, it was taken back to our field lab within 10 minutes. To reduce handling stress, which might affect the testosterone level, every captured grouse was placed in a black bag for half an hour [33]. Then 1–1.5 ml of blood was collected from the brachial vein and plasma separated in a centrifuge for 10 min at 30,000 rpm and kept frozen at −40°C for subsequent analyses. Plasma levels of testosterone were measured by radio-immunoassay (RIA) using the RIA Iodine [^125^I] Testosterone Radioimmunoassay Kit (Beijing Chemclin Biotech Co., Ltd.). First, 100 µL of standard or sample serum, 200 µL of ^125^ I-labeled testosterone derivative in buffer, and 200 µL of testosterone antiserum in buffer was pipetted into each tube, then mixed and incubated for 60 min at 37°C. To separate bound from free ^125^I-labeled testosterone, 1000 µL of (donkey anti-rabbit/PEG solution) was added to each tube and the contents were mixed. After an additional 15 min incubation at room temperature, the tube was centrifuged at 3600 rpm for 20 min, the supernatants were decanted, and the radioactivity in the precipitate (CPM) was counted [34], [35]. Then the standard curve was constructed for estimating plasma levels of testosterone (Figure S1). The minimum detectable dose in the assay was 20 pg/ml, with intra-assay and inter-assay coefficients of variance of 10% and 15%, respectively. Plasma levels of testosterone were measured in duplicate samples in a single assay, the values being highly and significantly repeatable; *F*_1,35_ = 18.254, *r* = 0.944, *P*<0.001), and average testosterone values were used for the analysis.

Lutein is the most abundant circulating carotenoid in bird combs [36], HPLC and spectrophotometry are two common methods used for measuring lutein concentration [37]. Total plasma carotenoid concentration (µg/ml) was calculated in our experiment using a standard curve for lutein by spectrophotometry (Sigma Chemicals, catalogue No. X6250). Carotenoids were quantified by diluting 60 µl of plasma in acetone (1∶10 dilution). The mixture was vortexed and centrifuged at 10,000 rpm for 10 min to precipitate proteins. The supernatant was examined in a Shimadzu UV-1240 spectrophotometer and we determined the optical density at 446 nm, the wave length of maximal absorbance for lutein (Figure S2) [38]. Lutein was also measured in duplicate samples in a single assay, with values being highly and significantly repeatable (*F*_1,35_ = 0.12, *r* = 0.99, *P*<0.01), and average values were used for the analysis.

### Color Measurements

After drawing blood, we took standardized digital photos of each bird in a dark room with a flashlight at a constant distance of 40 cm (Camera model: Canon EOS 20D), with a white wall as background and a ruler with red, yellow, and black reference stripes as described on the website www.ebd.csic.es/rv/index.html, downloaded at 11-21-2007. Following previous studies with this color measurement in carotenoid-pigmented species, the intensity of carotenoid-based red coloration (redness hereafter) was calculated as R divided by the average of R, G and B [39], [40]. Because comb color varied with illumination, we adjusted the values of R, G, and B using the reference chips placed in all comb photos following previously described methods (Text S1) [41]. To estimate the repeatability of comb color, the same grouse were photographed twice and each photo was used to calculate the redness twice; the values were highly and significantly repeatable within photos (*F*_1,47_ = 4.17, *r* = 0.99, *P*<0.01) and between photos (*F*_1,47_ = 524.42, *r* = 0.94, *P*<0.01).

### Biometrical Data

**Figure 1:**
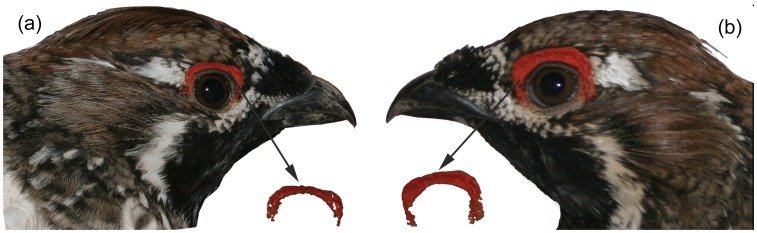
Comparing the supra-orbital comb between mated and unmated male Chinese grouse (a, unmated male grouse; b, mated male grouse).

For all captured grouse, we measured body mass (to the nearest 5 g, using a 1 kg Pesola spring balance), plus wing length, tail length, and bill length (all to the nearest 1 mm) following standard procedures [42], [43]. Tail length: the distance from the base to the tip of the longest tail feathers (rectrices). Wing length: the distance from the distal portion of the carpus to the tip of the longest primary feather. Bill length: straight line from bill tip to feathering at base of bill. Pectoral muscles of the grouse were closely correlated with body weight, and reflect variation in reserves of protein and possibly intramuscular fat estimated. Studies on the grouse found that ‘plumpness’ of the pectoral muscles was correlated to keel angle [44]. Thus, the index of body condition was scored according to the plumpness of the pectoral muscles. Keel angle was measured perpendicular to the keel 2 cm from the anterior edge: 1) The bird was placed horizontally on its back, and a compass was placed on the skin and carefully pressed evenly across the chest on both sides of the keel simultaneously. 2) Then the compass was placed flat on a piece of paper and the shape of the angle drawn with a pencil directly onto the paper. 3) The angle between two legs of the compass points situated 2 cm was measured with a protractor. Each Chinese grouse was measured twice by the same researcher (Yang), with the keel angle of the grouse ranging from 42 to 96 (means = 67.63±1.89, n = 35), with the results being highly and significantly repeatable (*F*_1,34_ = 8.40, *r* = 0.97, *P*<0.01). Then, the average keel angle of the two measurements was divided into 5 classes by K-means Cluster: 1 (skeletal), 2 (relative thin), 3 (normal), 4 (relative fat), 5 (strong) [45]. Comb size was measured using Adobe Photoshop CS5 (Text S1). The photo scale and number of pixels within the comb area were used to calculate comb area, then the area of the comb in each image was calibrated by the measurement scale in mm^2^ (Figure 1) [12], [36]. The repeatability of comb size was also estimated within the same picture and between two different pictures of the same combs taken at the same time. Comb sizes were highly and significantly repeatable between photos (*F*_1,47_ = 6.16, *r* = 0.94, *P*<0.05) and within photos (*F*_1,47_ = 4.23, *r* = 0.81, *P*<0.05).

### Parasite Counts

*Trichostrongylus tenuis* is a common parasite in grouse in Europe, and it was first found in 1981 in the Zhejiang province, China. After dissecting a sick dead Chinese grouse, this nematode was found in the alimentary canal and their laid eggs were also found in faecal droppings (unpublished data). Faecal egg concentrations (eggs/gram faeces) provided reliable estimates of number of worms per grouse, so we used faecal egg counts for the level of the infection [46]. For each grouse, a 0.2 g sample of faeces was diluted in 5 ml of saline water, mixed thoroughly, placed on a MacMaster slide, and examined under 40X magnification. Faecal eggs were counted twice (values highly and significantly repeatable; *F*_1,35_ = 45.93, *r* = 0.99, *P*<0.01) and the average number of eggs used for later analyses.

### Home Range Size and Territorial Call Rate

All radio-tagged grouse were located daily from April to June. The positions of Chinese grouse were located using triangulation. We used incremental area analysis (IAA) to determine the minimum number of locations required for an individual’s home range to stabilize [47]. Minimum number of locations to calculate the home range size of each grouse varied with its spatial distribution of telemetry positions, and only grouse whose home range size became asymptotic given their number of locations were included in our analyses. IAA for 35 male grouse showed that the mean minimum number of locations for each grouse was 74.91±15.80 (n = 35), ranging from 31 to 91, so the necessary locations for each grouse was 91 and a total of 3185 locations were used to calculate home range size using the minimum convex polygon (MCP) method [48], [49], by software Ranges7eXtra v1.8 (Anatrack LTD. Wareham, UK). Males were classified as mated grouse if they accompanied the same female for a period of at least 10 days. Observing from a distance of 50–100 m and using standard sampling times of 10 min on mornings (06∶00–09∶00) [50], we recorded the frequencies of “flutter jumping” and “territorial flights” [51]. Total sampling time for behavior record was 455 hours, with each male of 780.01±246.06 min (n = 35), ranging from 330 to 1080 min. Observations began the day following capture and handling and continued for at least one month.

### Statistical Analyses

Because the testosterone level, condition score, redness, comb size and faecal egg counts were measured twice for each captured grouse, we used repeated measures analysis [52]. Generalized Linear Model (GLM) was used to test whether variation in sexual traits were explained by sex or, in males, by mating status. Because most variables varied with year, age and sampling date, we controlled sampling year, age and sampling date as fixed effects in models. For correlation analyses, partial correlation was used to control the age, sampling year, sampling date effect; Spearman correlation was used when the variable was not normally distributed. Continuous variables were tested for normality using Wilk-Shapiro tests and parasite counts were natural *ln* transformed to meet the assumption of normality. The method for post-hoc tests Fisher’s least significant difference (Fisher’s LSD). All results were shown as mean ± SE and all tests were two-tailed. Above statistical analyses were performed with SAS (Version 9.1, 2002).

## Results

### Comb Size, Color, Carotenoid and Testosterone Levels

Of the 35 males captured and marked, 21 had mates and 14 did not. Testosterone concentrations were explained by age (GLM: *F*_1, 34_ = 6.51, *P* = 0.02) and sampling date (GLM: *F*_23, 34_ = 4.10, *P* = 0.02), but not by sampling year (GLM: *F*_1,34_ = 1.07, *P* = 0.31). The mean testosterone concentrations in mated and unmated males were 103.82±11.93 pg/ml (n = 21), and 46.99±7.47 pg/ml (n = 14) respectively. After controlling for age and sampling date, mated males had significantly higher testosterone than unmated males (GLM: *F*_1,34_ = 13.27, *P*<0.01).

**Figure 2:**
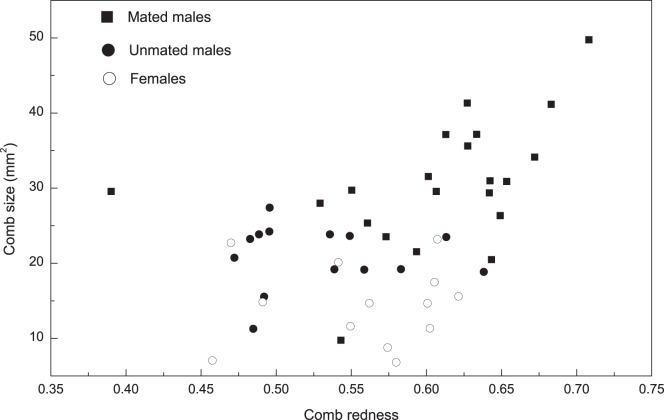
The correlation between comb size and color within the female, mated male and unmated male Chinese grouse.

Controlling for age, sampling year and sampling date, GLM analysis showed that significant differences existed among females, mated males, and unmated males with respect to body mass (*F*_2,47_ = 8.92, *P*<0.01) and comb size (*F*_2,47_ = 34.973, *P*<0.01). Similarly, significant differences were found in the redness of their combs (*F*_2,47_ = 3.51, *P*<0.05; Table 1). Multiple comparisons tests indicated, however, that the in body mass among these groups was not explained by male mating status (Fisher’s LSD: mean difference = 18.04, *P = *0.15), but the females were heavier than the males. In contrast, for comb size, the multiple comparisons test showed that the mean difference between mated and unmated males was highly significant (Fisher’s LSD: mean difference = 9.64, *P*<0.01). Mated males had bigger combs than unmated males, and both had bigger combs than females (Independent samples T test: *t*_33.9_ = 5.89, *P*<0.01). For comb color, a multiple comparisons test similarly showed mated males had redder combs than unmated males (Fisher’s LSD: mean difference = 0.08, *P*<0.01). The correlation between comb size and redness was not significant in unmated males (Spearman’s rank correlation, *r* = −0.01, *P*>0.05, n = 14) and females (Spearman’s rank correlation, *r* = 0.17, *P*>0.05, n = 13), but comb size were positive correlated to redness in mated males (Spearman’s rank correlation, *r* = 0.48, *P*<0.05, n = 21) (Figure 2). The correlation between testosterone and comb size was significant (Spearman’s rank correlation, *r* = 0.66, *P*<0.01, n = 35; Table 1) as was the correlation between testosterone and redness (Spearman’s rank correlation, *r* = 0.51, *P*<0.01, n = 35).

**Table 1:** Correlation between testosterone level and sexual traits and the comparison of sexual traits between mated and unmated males and female Chinese grouse in the Lianhuashan Nature Reserve, central China.

Male traits	Unmated males	Mated males	Females	*F*	*P*	Testosterone	
	(n = 14)	(n = 21)	(n = 13)			*r*	*P*
Body mass (g)	313.000±6.665	324.048±4.045	342.091±15.15	8.916	<0.01	−0.016	0.928
Comb size (mm^2^)	21.109±1.838	30.548±1.487	14.418±1.894	34.973	<0.01	0.661	<0.001
Comb Redness	0.532±0.016	0.608±0.013	0.557±0.016	3.506	<0.05	0.506	0.002
Wing length (cm)	14.011±0.117	12.934±1.974	15.001±1.215	0.535	>0.05	0.016	0.928
Tail length (cm)	14.091±0.094	14.224±0.146	13.450±0.368	0.002	>0.05	0.051	0.77
Bill length (cm)	1.948±0.023	1.976±0.033	1.887±0.066	0.125	>0.05	0.1	0.573

Note: General Linear Model (GLM) for the analysis, sampling year, sampling date and age were as fixed effects.

**Figure 3:**
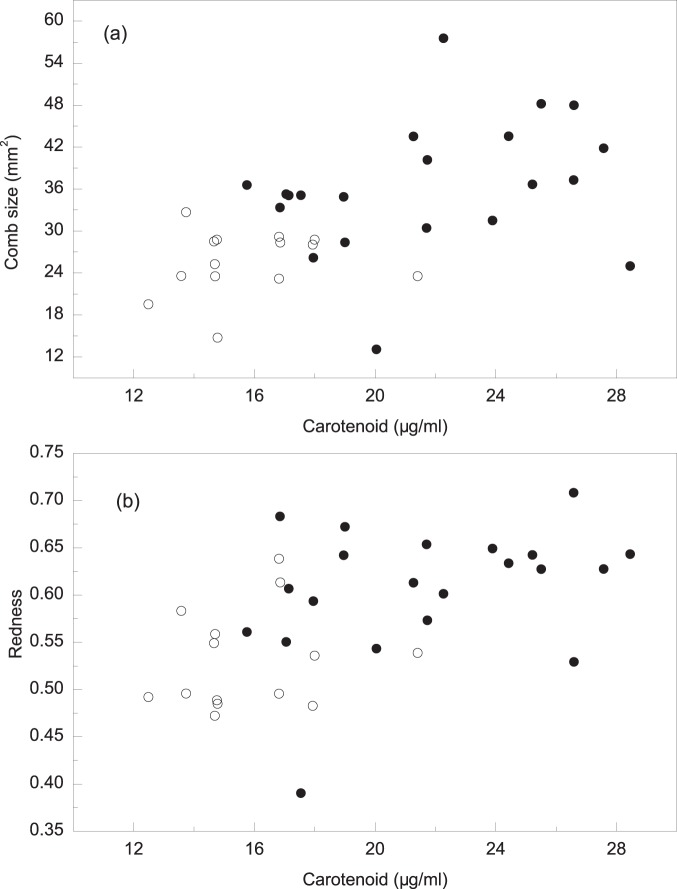
The correlation between plasma carotenoid concentration of male Chinese grouse and (a) comb size and (b) comb redness. Mated males (closed circles), Unmated males (open circles).

Carotenoid concentrations were significantly greater in mated males (21.69±3.98 µg/ml; n = 21) than in unmated males (Independent T test: 15.81±2.32 µg/ml, n = 14; *t*_33_ = 4.98, *P*<0.01). They were also significantly correlated with comb size (Spearman’s rank correlation, *r* = 0.54, *p*<0.01, n = 35; Figure 3a), and with redness (Spearman’s rank correlation, *r* = 0.53, *P*<0.01, n = 35; Figure 3b).

### Body Condition and Parasite Burdens

**Figure 4:**
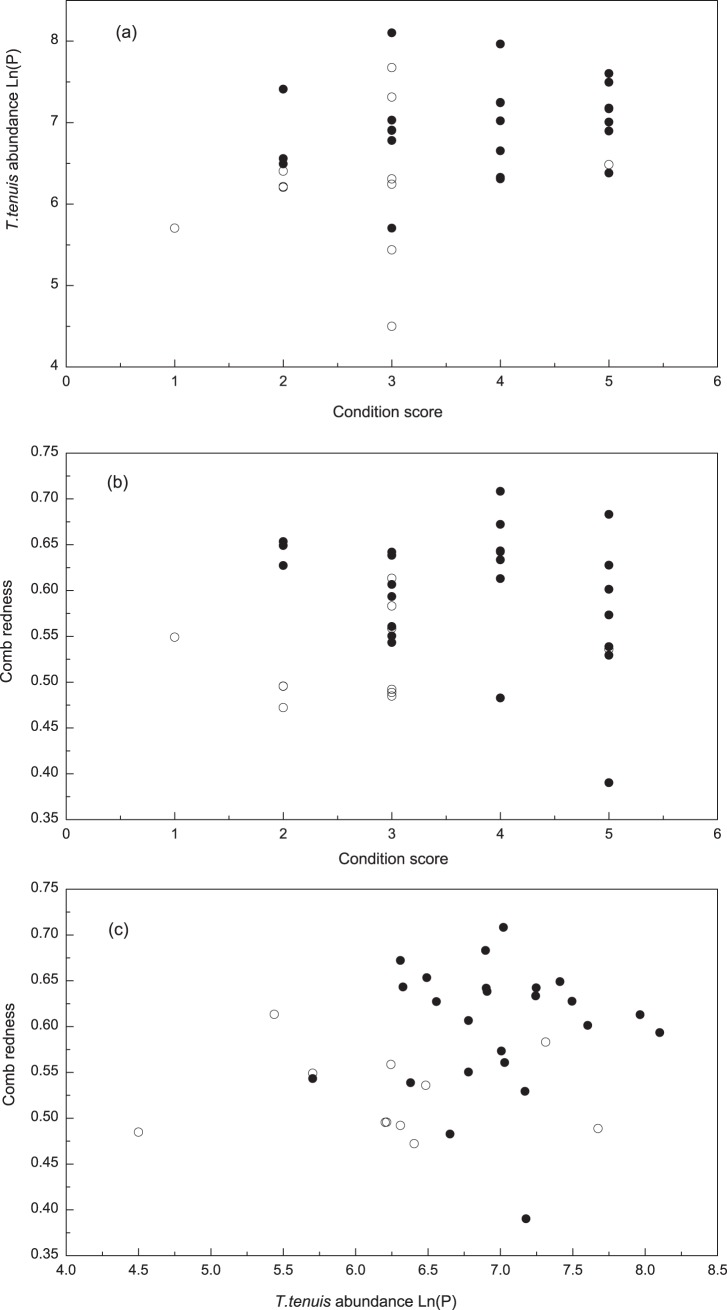
The correlations between comb redness, condition score and parasite burdens of male Chinese grouse. Mated males (closed circles), Unmated males (open circles).

Variation in parasite abundance ranged from 500 to 2833 eggs per grouse, a range not significantly explained by age (GLM: *F*_1,34_ = 3.96, *P* = 0.06), or year (GLM: *F*_1,34_ = 1.86, *P* = 0.18). Parasite abundance (*lnP*) was significantly greater in unmated males than in mated males (GLM: *F*_1,34_ = 6.57, *P*<0.05). Parasite burden was negatively correlated with condition score (Partial correlation, *r* = −0.344, *P*<0.05, n = 35), and positive with testosterone (Partial correlation, *r* = −0.98, *P*<0.01, n = 35). That is, males with higher condition score and testosterone had fewer parasites and redder combs (Figure 4).

### Home Range and Territorial Behavior

**Figure 5:**
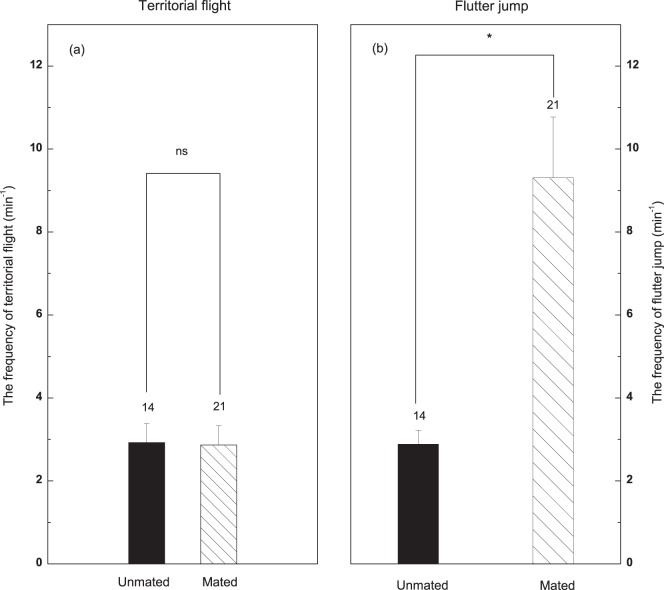
Behavioral comparisons of mated and unmated male Chinese grouse (a) territorial flights, (b) flutter-jumps. The numbers above the bars are sample sizes.

**Figure 6:**
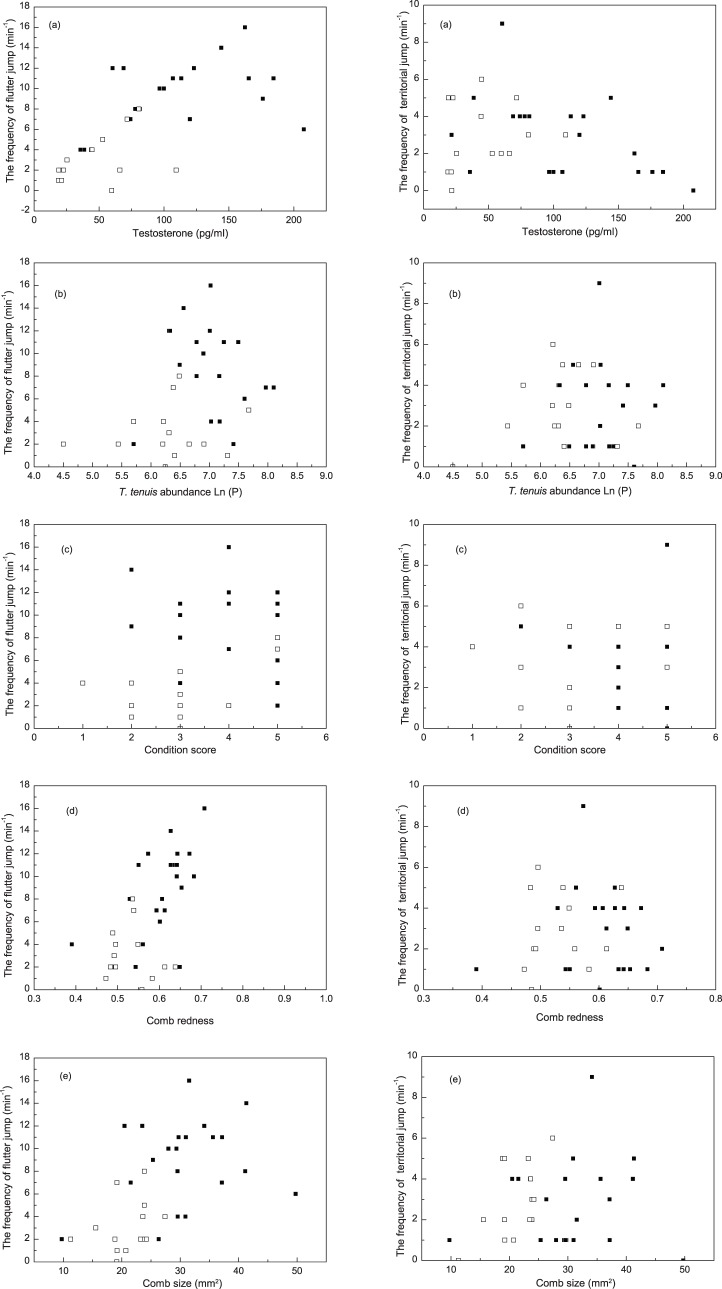
The correlations between territorial behaviors of male Chinese grouse (territorial flights, flutter-jumps) and assorted variables (a, testosterone; b, *T. tenuis* abundance; c, condition score; d, comb redness; e, comb size). Mated males (closed squares), Unmated males (open squares).

The mean home range size of mated males (9.05±1.54 ha, n = 21) was larger than that of unmated males (6.55±1.50 ha, n = 14), but not significantly so **(** *t*_33_ = 1.03; *P* = 0.31). The frequency of territorial flights was almost identical in mated and unmated males (Independent samples T test: *t*_33_ = −0.09; *P* = 0.93; Figure 5a), but mated males performed significantly more flutter jumps (Independent samples T test: *t*_33_ = 2.62; *P*<0.05; Figure 5b). Territorial flights were positively correlated to testosterone (Partial correlation, *r* = 0.34, *P*<0.05, n = 35), but not correlated with condition score (Partial correlation, *r* = 0.01, *P* = 0.97, n = 35), parasite (Partial correlation, *r* = −0.19, *P* = 0.29, n = 35), comb size (Partial correlation, *r* = −0.18, *P* = 0.32, n = 35), or redness (Partial correlation, *r* = −0.08, *P* = 0.65, n = 35). Frequency of flutter jumps was correlated with testosterone (Partial correlation, *r* = 0.37, *P*<0.05, n = 35), parasite (Partial correlation, *r* = −0.71, *P*<0.01, n = 35), comb size (Partial correlation, *r* = 0.37, *P*<0.05, n = 35), and redness (Partial correlation, *r* = 0.43, *P*<0.05, n = 35, but not correlated to condition score (Partial correlation, *r* = 0.19, *P* = 0.28, n = 35) (Figure 6).

## Discussion

### Comparison on the Methods for Measuring Color

Sexually selected traits in birds, such as comb, feather, bill, eye ring, were colorful and may play a key role in avoiding predators, attracting mates and species recognition. However, the color patterns for these traits were not the same and were mainly divided into 2 categories: structural and pigment dependent coloration. In contrast to those produced by pigments, the structural color arises from the physical interaction of light with biological nanostructures and rely on the shape of the material, not its chemical properties, so electron microscope and spectrophotometry are good tools for exploring the surface ultrastructure of selected traits [53].

Pigment dependent coloration relies on molecules that produce colors by the selective absorption and reflection of specific wavelengths of electromagnetic radiation, so reflectance spectrophotometry and digital camera are two common instrumental approaches for measuring the pigment color. The principle of spectrophotometry is based on the measurement the reflectance form or the transmittance through materials as a function of wavelength, so it is more precise, but also costly and time-consuming. Furthermore, spectrophotometry may lead to systematic errors if the surface of the object is uneven (spatial variation). The use of digital cameras for quantifying pigment color can image each pixels of the object surface and calculate the values of three color pigments, red, green and blue (RGB). But camera-based color measurement varied with ambient illumination, it may affect the repeatability of color score within photos and between photos. Setting a reference band in the experiment could ensure this method to meet the requirement of accuracy and anti-interference, so camera-based digital imaging could be a reliable method for measuring pigment color [41]. In the field, non-contact camera-based digital imaging systems are much preferred, due to being rapid, effective and noninvasive.

### Are Comb Size and Color Honest Signals of Male Quality in Chinese Grouse?

Previous research has suggested that contestants for valuable resources should escalate agonistic encounters from cheap, general signals of resource-holding potential to more costly and more accurate ones (*i.e.* difficult to falsify) [54]. Furthermore, natural selection should favor honest advertisements of resource-holding potential because both participants in such systems (*i.e.* signal sender and signal receiver) reduce their risk of unnecessarily engaging in dangerous fighting behaviors [55].

Avian ornamentation such as combs, lores, ceres, bills, plumage color and tail length are influenced by sex-steroids [8], [9], but being for the most part inert and no longer vascularized, they cannot respond to short-term hormonal fluctuations [56]. Only combs, wattles and eye rings lend themselves to honest signaling of current body condition and value as a breeding partner [11], [56]. For example, the redness of the red grouse combs is positively correlated with plasma testosterone concentration and artificially increasing testosterone levels rapidly enhances both comb redness and the level of circulating carotenoids, the latter effect resulting from a testosterone-mediated re-allocation of carotenoids from immune functions towards sexual ornamentation [23].

Recent experimental work on male barn swallows (*Hirundo rustica erythrogaster*) indicates that androgen concentrations are influenced by an individual’s signal expression, which rejects the prevailing view that costly physiological processes drive the sexual signal display unidirectionally [57]. Rubenstein and Hauber also pointed out that the relationships between sexual ornaments, reproductive behaviors and steroid hormones are bidirectional, they are dynamic feedbacks [58].

In our study, the grouse were captured randomly from March to June, with only 3 males captured before pairing. So we could not answer the question whether or not social status would affect the comb color and size due to the unequal and small sample size. As most of the grouse captured after pairing, we had to ignore the effect of sampling dates on changing the social selected traits. Although our data were not enough to explain how males would hormonally respond to intraspecific mating status (*i.e.* mating status feedback upon hormones, parasite), it should be still necessary for predicting the androgen patterns and their relationship with ornaments and mating status (*i.e.* hormonal regulation of mating status).

For the Chinese grouse we chose six biometrical indices as candidate signals of body condition but our results indicate that only comb size and color were significantly different between mated and unmated males and positively correlated with testosterone level (Table 1). Redness was also positively correlated to carotenoid concentration (Figure 2), with mated males therefore having higher concentrations than unmated males. We also found a positive correlation between body condition and comb redness. Recent research has shown that carotenoid absorption may depend on testosterone through its regulation of carotenoid-transporting lipoproteins in the bloodstream [59]. We consequently suggest that comb size and redness of male Chinese grouse are honest, testosterone-mediated signals revealing their body health, and thus play a crucial role in sexual selection in this species. Work on red grouse has shown that males with high testosterone have better breeding success [19] and we accordingly suggest that better breeding outcomes have selected for female Chinese grouse that preferentially mate with males endowed with better ornamentation that honestly signals their better body condition.

### The Relationship between Parasite Burden and Carotenoid-based Ornaments

An experiment in the red jungle fowl (*Gallus gallus*) showed that caecal nematodes may degrade sexual ornamentation through competition for carotenoids and through damage to the caecal mucosa that negatively affects carotenoid absorption [4]. The reduction of carotenoids in the bloodstream may in turn limit the brightness of carotenoid-based ornamentation such as combs or eye rings [23], [60]. For example, red grouse comb color was sensitive to manipulations of nematode load [61]. In addition, the same parasite may have other negative effects on body condition, energy intake, breeding, and survival [62]–[65].

In the Chinese grouse, we similarly found that *T. teniuis* abundance was negatively correlated with comb color and body condition. Thus, stronger individual males (i.e., those individuals able to obtain breeding opportunities) had smaller parasite burdens and larger and brighter combs (Figure 4a, c). A plausible reason for this relationship is that metabolic transformation of carotenoids requires energy and this can be ill-afforded by males in poor body condition [66], [67]. Females selecting males with enhanced ornamentation are therefore acquiring healthier mates with fewer parasites and both are benefiting from the honest male signals.

### Home Range, Territorial Behavior and Testosterone

The classic “territorial behavior” hypothesis in grouse proposed that testosterone levels would regulate male aggressiveness and that the associated territorial behavior would influence recruitment and population dynamics [68], [69]. This view fails to account for the negative effects of parasites on body condition or the risk of injury in increased territorial conflicts incurred by hyper-aggressive males [17]. Females mating with the males with more exaggerated ornaments may gain direct benefits such as essential territorial resources, paternal care or avoidance of infectious diseases.

In grouse exhibiting lek behavior (e.g., black grouse, *Tetrao tetrix*) high testosterone levels permit males to occupy the center of the lek and achieve high mating success [33]. In contrast, Chinese grouse are monogamous and they do not form leks, but instead attract females by defending high-quality territories. Because of the high population density of Chinese grouse in our study area and a limit of of high-quality territories available, territorial behavior was observed not only during the breeding season, but also through the autumn after the breeding season [70]. The frequency of flutter jumps, the major territorial defense behavior in Chinese grouse, was significantly greater in mated males (Figure 4). Plausibly, such birds, with higher testosterone levels and lower parasite loads, could acquire and maintain better territories than unmated males. Correlation analysis found that flutter jumps could be explained by testosterone, parasite, comb size and redness (Figure 6). Our results imply that females are able to nest and raise their offspring in a good habitat by mating with males with redder and larger combs, and reduce the risk of being infected by parasites by mating with the males with a reduced parasite load. Our results also provide experimental evidence for a direct benefit for the females mating with the males with more exaggerated ornament, however, we were unable to test the indirect benefit due to a lacking of data on the survival and reproduction of individuals and offspring.

Previous research on the blue grouse (*Dendragapus obscurus*) found that territory quality is associated with breeding success, but not with home range size [71]. In the Chinese grouse, good territories should have more food and cover and less risk of predation but not necessarily greater size [29]. Our results also showed that the difference in territory size between mated and unmated male Chinese grouse is not significant. We speculate that habitat quality may be the main limiting factor influencing breeding success in the Chinese grouse. The relationship between territory quality and sexually selected traits in the Chinese grouse will be addressed in future work.

## Supporting Information

Figure S1
**The standard curve for measuring testosterone concentration by method of RIA.** (1) To pipet 100 µL of standard or sample serum, 200 µL of ^125^I-labeled testosterone derivative in buffer, and 200 µL of testosterone antiserum in buffer into each tube, mix, and incubate for 60 min at 37°C. (2) To separate bound from free ^125^I-labeled testosterone, 1000 µL of (donkey anti-rabbit/PEG solution is added to each tube and the contents are mixed. (3) After a further 15 min incubation at room temperature, the tube is centrifuged at 3600 rpm for 20 min, the supernates are decanted, and the radioactivity in the precipitate (CPM) is counted. (4) The standard curve was constructed by plotting the testosterone concentration (log A) for each standard concentration on the abscissa (x) axis, and the value of B/B_0_ on the ordinate (y) axis. 
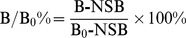
.(EPS)

Figure S2 **The standard curve for measuring carotenoid concentration by method of spectrophotometry.** (1) Carotenoids were quantified by diluting 60 µl of plasma in acetone (1∶10 dilution). The mixture was vortexed and centrifuged at 10,000 rpm for 10 min to precipitate proteins. (2) The supernatant was examined in a Shimadzu UV-1240 spectrophotometer and we determined the optical density at 446 nm, the wave length of maximal absorbance for lutein. And lutein concentrations determined based on chromatogram peak areas using an external standard of lutein prepared from marigold (Sigma Chemicals, USA, X6250). (3) The lutein stock solution was 100 µg/ml, which was prepared by dissolving 1.00 mg of lutein (Sigma, USA) in 10.00 ml acetone, then lutein diluted into 6 concentration gradients (100, 50, 25, 12.5, 6.25, 3.125 µg/ml). (4) Then graph our standard curve by plotting the lutein level (µg/ml) for each standard concentration (C) on the abscissa (x) axis, and the absorbance (A) on the ordinate (y) axis.
(EPS)

Table S1 **The information for captured grouse, including capture time, frequency of transmitter and mating status.** In the spring of 2008 and 2009, we captured 35 male Chinese grouse using snare poles, nets, or walk-in traps, and equipped with necklace transmitters (ID: the frequency of transmitter were used for mark each grouse).
(DOCX)

Text S1 **The procedure for quantifying comb color and calculating comb size by photography.**
(DOCX)
