# Supplementary material for: Is Sexual Ornamentation an Honest Signal of Male Quality in the Chinese Grouse (Tetrastes sewerzowi)?
Source: PLoS One. 2013 Dec 26;8(12):e82972. doi: 10.1371/journal.pone.0082972 (PMC3873284; doi:10.1371/journal.pone.0082972)
Supplement: Table S1 — The information for captured grouse, including capture time, frequency of transmitter and mating status. In the spring of 2008 and 2009, we captured 35 male Chinese grouse using snare poles, nets, or walk-in traps, and equipped with necklace transmitters (ID: the frequency of transmitter were used for mark each grouse). (DOCX) [file pone.0082972.s003.docx]

**Table S1. The information for captured grouse, including capture time, frequency of transmitter and mating status. In the spring of 2008 and 2009, we captured 35 male Chinese grouse using snare poles, nets, or walk-in traps, and equipped with necklace transmitters (ID: the frequency of transmitter were used for mark each grouse).**

| *Capture Date* | *ID* | *Mating status* | *Capture time before or after paring* |
| --- | --- | --- | --- |
| 2008/4/4 | 150.880 | No paired | After paring |
| 2008/4/5 | 150.862 | No paired | After paring |
| 2008/4/6 | 150.818 | Paired | After paring |
| 2008/4/7 | 151.431 | No paired | After paring |
| 2008/4/7 | 151.267 | Paired | After paring |
| 2008/4/8 | 150.899 | Paired | After paring |
| 2008/4/11 | 151.110 | No paired | After paring |
| 2008/4/14 | 151.631 | Paired | After paring |
| 2008/4/16 | 151.071 | Paired | After paring |
| 2008/4/19 | 151.512 | Paired | After paring |
| 2008/5/9 | 151.390 | Paired | After paring |
| 2008/5/9 | 151.349 | No paired | After paring |
| 2008/5/9 | 151.370 | No paired | After paring |
| 2008/5/14 | 150.899 | Paired | After paring |
| 2008/5/17 | 150.322 | No paired | After paring |
| 2008/5/20 | 151.818 | Paired | After paring |
| 2008/5/24 | 151.231 | No paired | After paring |
| 2008/6/4 | 151.110 | Paired | After paring |
| 2009/3/29 | 151.032 | Paired | Before paring |
| 2009/3/30 | 151.862 | Paired | Before paring |
| 2009/3/31 | 150.431 | No paired | Before paring |
| 2009/4/1 | 150.267 | Paired | After paring |
| 2009/4/12 | 151.409 | No paired | After paring |
| 2009/4/12 | 151.466 | Paired | After paring |
| 2009/4/13 | 150.370 | No paired | After paring |
| 2009/4/21 | 151.940 | Paired | After paring |
| 2009/4/23 | 151.289 | No paired | After paring |
| 2009/4/24 | 151.311 | Paired | After paring |
| 2009/4/28 | 151.009 | No paired | After paring |
| 2009/4/29 | 151.867 | No paired | After paring |
| 2009/4/29 | 150.349 | Paired | After paring |
| 2009/5/2 | 151.328 | Paired | After paring |
| 2009/5/3 | 151.322 | Paired | After paring |
| 2009/5/6 | 151.726 | Paired | After paring |
| 2009/5/9 | 151.750 | Paired | After paring |
