# Supplementary material for: Is Sexual Ornamentation an Honest Signal of Male Quality in the Chinese Grouse (Tetrastes sewerzowi)?
Source: PLoS One. 2013 Dec 26;8(12):e82972. doi: 10.1371/journal.pone.0082972 (PMC3873284; doi:10.1371/journal.pone.0082972)
Supplement: Text S1 — The procedure for quantifying comb color and calculating comb size by photography. (DOCX) [file pone.0082972.s004.docx]

**Text S1. The procedure for quantifying comb colour and calculating comb size by Photography.**

1. Quantifying Colour

We took standardized digital photos of each bird in a dark room with a flashlight at a constant distance of 40 cm, with a white wall as background and a ruler with red, yellow, and black reference stripes. The protocol for photography was similar to that described previously research. Because comb color varied with illumination, we adjusted our results using the calibration stripes following Villafuerte and Negro (1988), under the equation *y= a + bx* (where *y* is the expected value for instance of the "R", and *x* its corresponding expected value after transforming).

(1) We calculated the average of values of R, G, B for two reference chips in each photo. (2) The coefficient (*b*) and intercept (*a*) could be calculated by the two reference chips. Then the values of R, G, B of the comb were standardized by the reference chips to control the variation of the illustration between different photos.

For each pixel within the area of visible nuptial coloration red (*r*), green (*g*) and blue (*b*) values in RGB colour-space were extracted, averaged for the whole patch and a single redness intensity value (*R*) calculated as:

.

*R* therefore represents the red component of the selected pixels, with larger values indicating redder patches.

2. Calculating comb size

The comb of Chinese grouse is irregular polygon, we calculate the comb size by Adobe Photoshop CS5.

(1) The comb was selected by the tool of magic stick;

(2) Number of pixels within comb could be recorded in the information window;

(3) Comb size in each image was calibrated by the measurement scale (correction factor).

The equation for calculating comb size is as the following:

$$\text{Comb size=}\frac{\text{Photo length }\times\text{ Photo width }\times\text{ Number of pixels within comb}}{\text{Number of pixels within photo}}\times\text{Correction factor}$$
